# Sophoricoside ameliorates cardiac hypertrophy by activating AMPK/mTORC1-mediated autophagy

**DOI:** 10.1042/BSR20200661

**Published:** 2020-11-19

**Authors:** Maomao Gao, Fengjiao Hu, Manli Hu, Yufeng Hu, Hongjie Shi, Guo-Jun Zhao, Chongshu Jian, Yan-Xiao Ji, Xiao-Jing Zhang, Zhi-Gang She, Hongliang Li, Lihua Zhu

**Affiliations:** 1 Medical Research Institute, Wuhan University, Wuhan 430072, China; 2 Medical Science Research Center, Zhongnan Hospital of Wuhan University, Wuhan 430071, China; 3 Institute of Model Animal of Wuhan University, Luojia Mount Wuchang, Wuhan 430072, China; 4 Department of Cardiology, Renmin Hospital of Wuhan University, Wuhan 430060, China; 5 Basic Medical School, Wuhan University, Wuhan 430071, China

**Keywords:** AMPK, autophagy, cardiac hypertrophy, Sophoricoside

## Abstract

**Aim**: The study aims to evaluate protective effects of sophoricoside (Sop) on cardiac hypertrophy. Meanwhile, the potential and significance of Sop should be broadened and it should be considered as an attractive drug for the treatment of pathological cardiac hypertrophy and heart failure.

**Methods**: Using the phenylephrine (PE)-induced neonatal rat cardiomyocytes (NRCMs) enlargement model, the potent protection of Sop against cardiomyocytes enlargement was evaluated. The function of Sop was validated in mice received transverse aortic coarctation (TAC) or sham surgery. At 1 week after TAC surgery, mice were treated with Sop for the following 4 weeks, the hearts were harvested after echocardiography examination.

**Results**: Our study revealed that Sop significantly mitigated TAC-induced heart dysfunction, cardiomyocyte hypertrophy and cardiac fibrosis. Mechanistically, Sop treatment induced a remarkable activation of AMPK/mTORC1-autophagy cascade following sustained hypertrophic stimulation. Importantly, the protective effect of Sop was largely abolished by the AMPKα inhibitor Compound C, suggesting an AMPK activation-dependent manner of Sop function on suppressing pathological cardiac hypertrophy.

**Conclusion**: Sop ameliorates cardiac hypertrophy by activating AMPK/mTORC1-mediated autophagy. Hence, Sop might be an attractive candidate for the treatment of pathological cardiac hypertrophy and heart failure.

## Introduction

Heart failure is defined as the inability of heart to supply the peripheral tissues with the required amount of blood and oxygen to meet their metabolic demands, and is an epidemic disease in the modern world affecting approximately 1–2% of adult population [1]. Meanwhile, heart failure is a syndrome with symptoms and signs caused by cardiac dysfunction, resulting in reduced longevity [2]. The pathogenesis of heart failure is complex and diverse, which leads to little effect of current interventions.

Heart failure is usually accompanied by cardiac hypertrophy and fibrosis [3]. Under numerous hypertrophic stimuli, such as chronic inflammatory response, pressure overload, ischemia, valvular heart disease and excess neurohormonal activation, cardiac hypertrophy eventually develops into maladaptive cardiac remodeling and heart failure [4]. The main pathological features of cardiac hypertrophy includes cardiomyocyte enlargement, overexpression of fetal genes, protein synthesis increase, myocardial fibrosis, cell signal dysfunction and autophagy inhibition [5,6]. A great deal of signaling pathways have been well validated in the progression of cardiac hypertrophy [7]. Therefore, pharmacological therapies that modulate these signaling pathways might embody great promise for treating pathological cardiac hypertrophy.

Cardiomyocyte is finely orchestrated upon extracellular challenge via a complex molecular network, where autophagy functions as a key switcher from compensation to decompensation during a chronic stimulation [6]. In terms of cardiac hypertrophy, autophagy has been reported to alleviate intracellular oxidative injury and maintain cardiac function by maintaining ATP level [8]. Under normal conditions, low level of constitutive autophagy is essential for cell growth, development and homeostasis, while either insufficient or excessive autophagy triggers cell death, indicating dual roles of autophagy in both pathological and physiological conditions [9]. Genetic or pharmacological interventions of autophagy could reverse pressure overload-induced cardiac remodeling and contractile dysfunction [10]. These findings elucidate that targeting autophagy might represent a promising therapeutic strategy for pathological cardiac hypertrophy.

Sophoricoside (Sop) is an isoflavone isolated from the dried fruit of *Sophora japonica* L. [11]. Since first isolation and identification, Sop is gradually known to possess the capacity of protecting against fertility, tumor and dermatitis [11–13]. Sop plays an important role in inhibiting inflammatory response. For example, Sop improved the acute and chronic contact dermatitis by blocking NF-κB signal activation in B cells [14]. In human mast cells, Sop could also inhibit the production of inflammatory cytokines [15]. Meanwhile, Sop inhibited lipid accumulation in HepG2 cells and stimulated glucose consumption in C2C12 myotubes in cases of obesity and type II diabetes [12]. Sop also protected against fructose-induced liver injury via regulating lipid metabolism, oxidation in non-alcoholic fatty liver disease [11]. These results revealed potent pharmacological effects of Sop in the treatment of various diseases. However, the role of Sop in pathological cardiac hypertrophy remains unknown.

In the present study, we examined the anti-cardiac hypertrophy potential of Sop in *vitro* and in *vivo*. At first, Sop administration significantly mitigated cardiomyocyte enlargement, improved cardiac hypertrophy and suppressed a long-term heart remodeling. Further investigations of molecular mechanisms revealed that the prevention of pathological myocardial hypertrophy by Sop depends largely on the activation of AMPK/mTORC1-mediated autophagy.

## Methods

### Mouse studies

All the experiments that involved animals were approved by the Animal Care and Use Committee of Renmin Hospital of Wuhan University and were organized in accordance with the National Institutes of Health Guide for the Care and Use of Laboratory Animals. All animals received humane care and the acclimatization period was at least 1 week.

### Animal feeding and environmental conditions

All the experimental mice were raised in the SPF level of Animal Experiment Center of Wuhan University. They were alternately illuminated for 12 h per day, with temperature of 24 ± 2°C, humidity of 40–70%, and free feed and water.

### Transverse aortic constriction

The pressure overload-induced cardiac hypertrophy model in mice was established via transverse aortic constriction (TAC) surgery. The male mice weighing 25.5–27 g (8–10 weeks) were subjected to TAC or sham operation as previously described [16]. All operations were conducted under sterile conditions and anesthetized by intraperitoneal injection of pentobarbital sodium inhalation (Sigma–Aldrich #P3761, St. Louis, Missouri, U.S.A.). Then, mice were subjected to a midline incision to expose the aortic arch. The aortic arch was banded against 26-G needle with 7-0 silk suture. The needle was subsequently removed before abdominal closure. Similar procedures without aortic constriction were conducted in the sham group mice.

### Intragastric administration of Sop

Sop (80 or 160 mg/kg per day dissolved in 0.9% NaCl) was continuously gavaged to TAC group mice for 4 weeks. TAC or sham surgery treated mice that were subjected to the same volume of Saline served as infusion controls.

### Echocardiographic measurements

Echocardiography measurements were implemented at the indicated times to evaluate the cardiac function of the mice [17,18]. First, the surviving mice were anesthetized using isoflurane (1.5–2%). For echocardiography measurements, M-mode tracings derived from the short axis at the papillary muscle level were recorded using a Small Animal Ultrasound Imaging System (VEVO2100, FUJIFILM VISUALSONICS, Canada) equipped with a 30-MHz probe (MS400). In M-mode echocardiography, left ventricular volume and wall thickness were measured in three consecutive cycles. Then we measured the left ventricular end-systolic diameter (LVESd) and the left ventricular end-diastolic diameter (LVEDd). LV fractional shortening (LVFS) was calculated using the following formula: LVFS = (LVEDd - LVESd)/LVEDd × 100%

### Histological analysis

Five weeks after the induced cardiac hypertrophy model, animals were anesthetized with 80 mg/kg.bw pentobarbital sodium by intraperitoneal injection. Many indicators were recorded, such as body weight, heart weight (HW), lung weight, and tibial length. The hearts were fixed in 10% formalin and embedded in paraffin by standard histological protocols [19]. Several sections (5-μm-thick) obtained from the mid-papillary muscle level of each heart were stained with Hematoxylin and Eosin (H&E) to assess histopathology or with Picrosirius Red (PSR) to evaluate collagen content. The cross-sectional areas (CSAs) of myocytes and fibrotic areas were measured using a digital image analysis system (Image-Pro Plus, version 6.0).

### Primary rat cardiomyocytes culture and phenylephrine stimuli

Primary cultures of neonatal rat cardiomyocytes (NRCMs) were performed as described previously [20,21]. NRCMs were isolated from 1–2 days old Sprague–Dawley rats. Briefly, cardiac cells were isolated in PBS containing 0.125% trypsin from the hearts of neonatal rats. Subsequently, NRCMs were purified by removing cardiac fibroblasts. Then, NRCMs were seeded in six-well culture plates coated with gelatin at a density of 3 × 10^5^ cells/well in Dulbecco’s modified Eagle’s medium (DMEM)/Ham’s F12 medium including 10% (v/v) FBS and penicillin/streptomycin for 24 h. Subsequently, NRCMs were cultured with a serum-free maintenance medium for another 12 h. Different concentrations of Sop (10 or 50 μM) were used in different treatment groups for 1 h before being co-incubated with phenylephrine (PE; 50 μM) for 48 h.

### Immunofluorescence analysis

The cell surface area of NRCMs was assessed by immunofluorescent staining. Briefly, NRCMs were treated with PE or PBS coincubated with Saline or Sop (10 or 50 μM) for 48 h. The cells were then fixed with 4% paraformaldehyde for at least 30 min, then saturated into PBS with 0.2% Triton X-100 for 5 min. Subsequently, slides containing cells were incubated with α-actinin antibody (A7811, Sigma, 1:100 dilution) at 4°C for overnight, then slides were incubated with fluorescent secondary antibody for 1 h. Subsequently, the slides were sealed with DAPI. Images were captured using OLYMPUS DP72 fluorescence microscope (model BX51TRF). The surface areas and the length and width of cardiomyocytes were measured using a digital image analysis system (Image-Pro Plus, version 6.0).

### Western blotting

Hearts tissues and NRCMs were first lysed in lysis buffer (20 mM Tris/HCl pH 7.5, 150 mM NaCl, 1 mM EDTA, 1% Nonidet P-40, 0.5% sodium deoxycholate and 0.1% SDS) containing the complete Protease Inhibitor (no. 04693132001, Roche) and PhosStop phosphatase inhibitor (no. 4906837001, Roche). Tissues or NRCMs were incubated on ice for 15 min, followed by centrifugation at 14000×***g*** for 30 min at 4°C. The protein concentrations were determined using the BCA Protein Assay Kit (Pierce). Then the lysates (50 μg) were resolved by SDS/PAGE (Invitrogen) and transferred on to PVDF membranes (Millipore). After blocking with 5% (w/v) skim milk for 1 h at room temperature, membranes were incubated with the primary antibodies overnight at 4°C. After incubation with the secondary antibodies for 1 h at room temperature, immunoblots were revealed by the ChemiDoc^™^ XRS+ (Bio-Rad Laboratories). The expression levels of proteins were normalized to GAPDH. Original gels of representative Western blot images relating to indicated figures was shown in Supplementary Figure S7. The information of corresponding antibodies was shown in Supplementary Table S1.

### Quantitative real-time PCR

TRIzol® reagent (Invitrogen) was applied to extract total RNA from cultured cardiomyocytes and heart tissues as provided by the manufacturer. Then, RNA was reverse-transcribed into cDNA using the Transcriptor First Strand cDNA Synthesis Kit (Roche). Quantitative real-time PCR (qRT-PCR) amplification was operated in the SYBR Green PCR Master Mix (Applied Biosystems). The expression levels of mRNAs were normalized to GAPDH. The information of primer sequences was shown in Supplementary Table S2.

### Statistical analysis

All data statistics of this subject were presented as the mean ± s.d. Student’s two-tailed *t* test was used to compare the means of two-group samples and a one-way analysis of variance (ANOVA) was applied for comparison of multiple groups, followed by the least significant difference (equal variances assumed) or Tamhane’s T2 (equal variances not assumed) tests. All statistical analyses were performed with Statistical Package for the Social Sciences (SPSS) 21.0. software. *P*- value less than 0.05 was considered significant.

## Results

### Sop inhibits PE-induced cardiomyocyte hypertrophy

**Figure 1:**
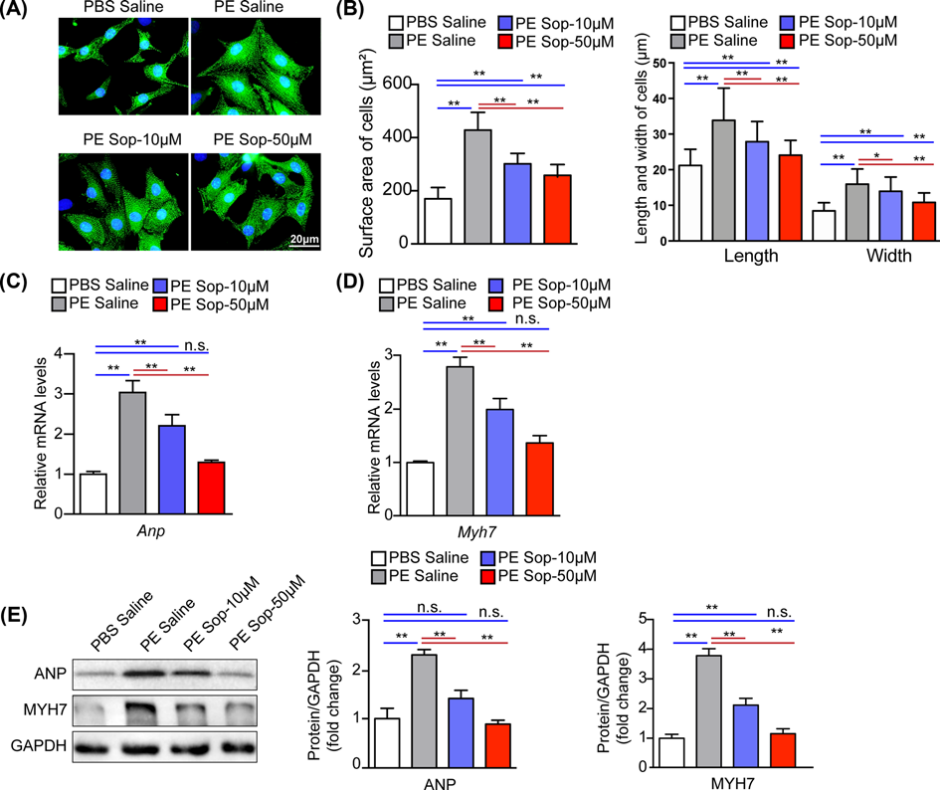
Sop inhibits PE-induced cardiomyocyte hypertrophy (**A**) Representative images of α-actinin (green) and DAPI (blue) stained NRCMs. Cells treated by PE or PBS in parallel and co-incubated with Sop or Saline treatment for 48 h, (scale bar, 20 µm), *n*=3 independent experiments. (**B**) Statistical quantification surface area of the NRCMs and length and width of NRCMs in the indicated groups, n=60 cells per group. (**C,D**) Real-time PCR analysis of mRNA expression levels of hypertrophic marker genes (*Anp, Myh7*), *n*=3 independent experiments. (**E**) Western blot analysis of protein levels and statistical quantification of the hypertrophy marker proteins (ANP, MYH7), *n*=3 independent experiments. The GAPDH level was used as the internal control in C–E. *, *P*<0.05; **, *P*<0.01; n.s., no significance, *P*>0.05.

To examine whether Sop protects cardiomyocytes against stress-induced hypertrophy, we isolated and subjected NRCMs to PE challenge, a well-recognized in *vitro* model to mimic cardiac hypertrophy. We first tested the role of Sop in cardiac hypertrophy. Immunofluorescence staining showed that Sop at different concentrations (10, 20, 50 μM) had no effect on cardiac hypertrophy (Supplementary Figure S1A,B), Meanwhile, the mRNA levels of cardiac markers A-type natriuretic peptide (*Anp*), myosin heavy chain 7 (*Myh7*)) had no changes after Sop treatment (Supplementary Figure S1C). Then, we treated NRCMs with PBS or PE and co-incubated with Saline or Sop at the indicated concentrations (10 or 50 μM) for 48 h. Results showed that PE induced a dramatic enlargement of NRCMs, which was effectively alleviated by treatment with Sop, as supported by quantified cell surface area and measurement of cell length and width of NRCMs (Figure 1A,B). Furthermore, the up-regulation of both mRNA levels and protein levels of hypertrophic marker genes (ANP, MYH7) triggered by PE was significantly blunted by Sop treatment (Figure 1C–E). These results indicate that Sop may be a negative regulator in PE-induced cardiomyocyte hypertrophy.

### Sop improves pressure overload-induced cardiac dysfunction

**Figure 2:**
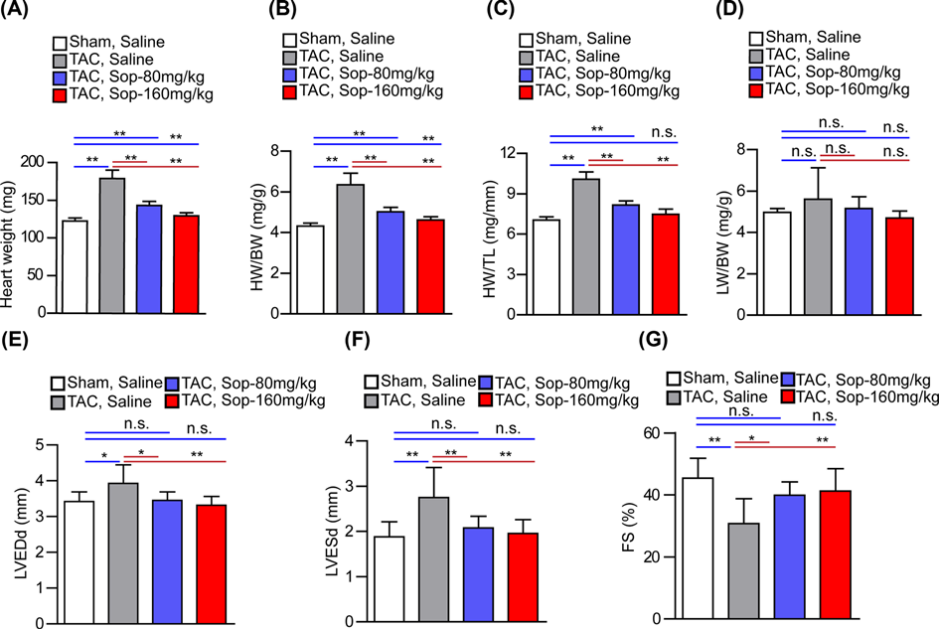
Sop improves pressure overload-induced cardiac dysfunction (**A–D**) Comparison of the (A) HW, (B) HW/BW, (C) HW/TL, and (D) LW/BW ratios in different treatment mice after sham or TAC surgery, *n*=10 mice per group. (**E**–**G**) Echocardiography results for the measurement of myocardial function (LVEDd, LVESd and FS) in the indicated groups, *n*=10 mice per group. *, *P*<0.05; **, *P*<0.01; n.s., no significance, *P*>0.05.

The function of Sop in cardiac hypertrophy and cardiac function was further detected *in vivo* based on pressure overload-induced mice model. One week after sham surgery, Sop was subjected to mice at 80 and 160 mg/kg/d or Saline for another 4 weeks. After 5 weeks of sham treatment, the ratio of HW to body weight (HW/BW), the ratio of lung weight to body weight (LW/BW), and the ratio of HW to tibia length (HW/TL) showed no significant change between Sham Sop group and Sham Saline group (Supplementary Figure S2A–C). To elucidate the function of Sop in cardiac hypertrophy, after TAC surgery, Sop solution was subjected to mice at 80 and 160 mg/kg/d for 4 weeks in mice. After 5 weeks of TAC surgery, pressure overload significantly induced cardiac hypertrophy, as evidenced by increased HW, HW/BW and HW/TL, compared with the sham Saline group. Remarkably, Sop treatment decreased HW, HW/BW and HW/TL ratios (Figure 2A–D). Furthermore, our echocardiography assay clarified that, after 5 weeks of sham surgery, LVEDd, LVESd and FS had no significant changement after Sop treatment, compared with the Sham Saline group (Supplementary Figure S2D–F). Compared with the sham cohorts, mice exhibited significantly increased chamber diameter and depressed systolic function and diastolic function after 5 weeks of TAC surgery as evidenced by increased LVEDd, LVESd and decreased FS. The heart dysfunction was effectively improved by treatment with Sop (Figure 2E–G).

### Sop alleviates pressure overload-induced cardiac hypertrophy

**Figure 3:**
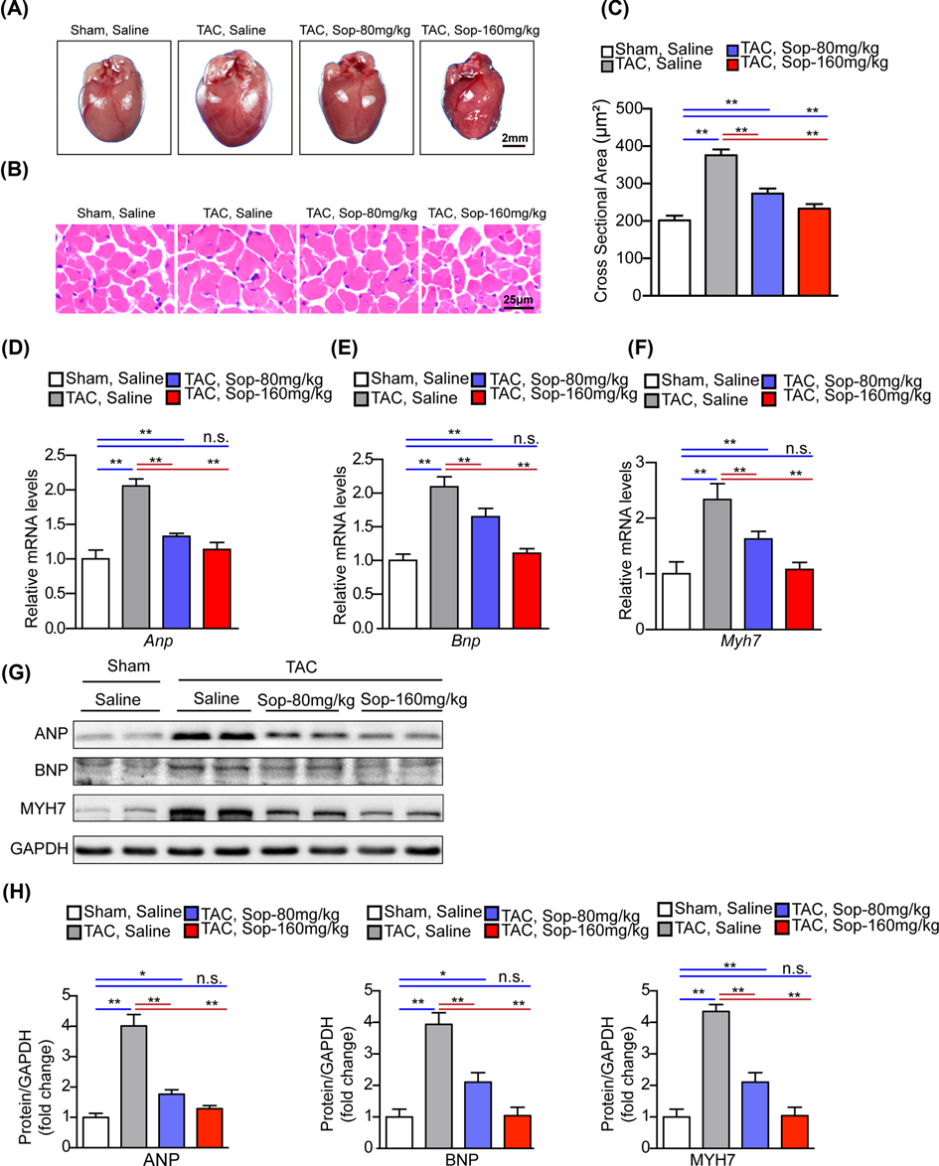
Sop alleviates pressure overload-induced pathological cardiac hypertrophy (**A**) Gross hearts from the indicated groups at 5 weeks after sham or TAC surgery, *n*=5-6 mice per group, scale bar, 2 mm. (**B**) Representative image of H&E staining from the indicated groups, *n*=6 mice per group, (scale bar, 25 µm). (**C**) Comparison of the CSA of cardiomyocytes from the indicated groups. (**D–F**) Real-time PCR analysis of mRNA expression levels of hypertrophic marker genes (*Anp, Bnp, Myh7*), *n*=4 mice per group. (**G,H**) Protein expression levels (**G**) and statistical quantification (**H**) of the hypertrophy marker proteins (ANP, BNP, MYH7), *n*=4 mice per group. The GAPDH level was used as the internal control in (D–H). *, *P*<0.05; **, *P*<0.01; n.s., no significance, *P*>0.05.

To further determine the effect of Sop on cardiac hypertrophy, we performed histological analysis from gross heart examinations and H&E staining. In line with the improved heart function, the visible heart enlargement was decreased by Sop treatment in the TAC mice (Figure 3A). Additionally, histological examination revealed decreased CSA of cardiomyocytes in Sop-treated, compared with the control group (Figure 3B,C). At the molecular level, TAC-induced up-regulation of hypertrophic markers including ANP, B-type natriuretic peptide (BNP) and MYH7, were strikingly suppressed by Sop treatment compared with vehicle controls (Figure 3D–H). Consistent with previous results, no significant changes were observed in the sham-operated mice treated with SOP (Supplementary Figure S3).

### Sop attenuates pressure overload-induced cardiac fibrosis

**Figure 4:**
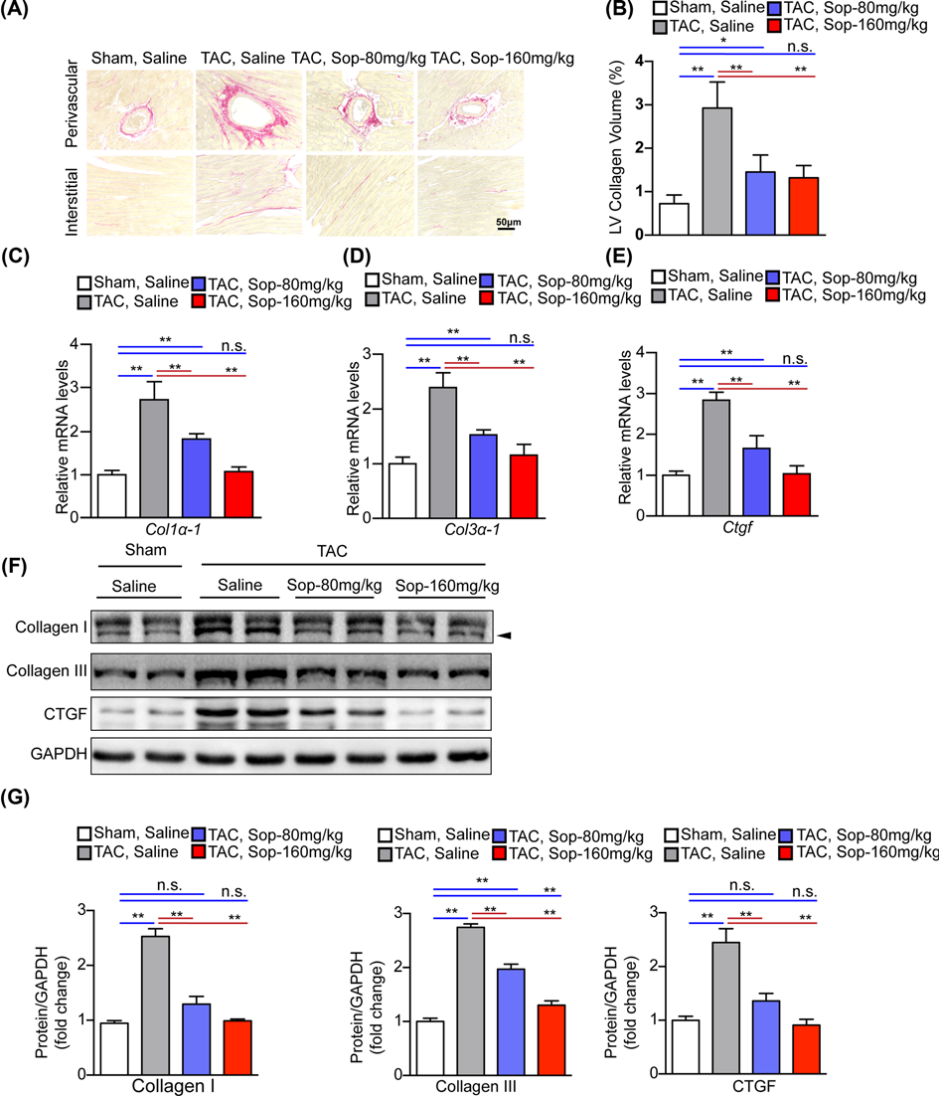
Sop attenuates pressure overload-induced cardiac fibrosis (**A**) Histological analyses of heart sections stained with PSR (scale bar, 50 µm) from the indicated groups 5 weeks after sham or TAC surgery, *n*=6 mice per group. (**B**) Comparison of the LV-collagen volume in the indicated groups. (**C**–**E**) Transcript levels of fibrotic marker genes (*col1a1, col3a1, ctgf*) in the indicated groups, *n*=4 mice per group. (**F,G**) Protein levels and statistical quantification of the fibrotic markers’ proteins (Collagen I, Collagen III and CTGF) in the indicated groups, *n*=4 mice per group. GAPDH was used as the internal control in (C–F). *, *P*<0.05; **, *P*<0.01; n.s., no significance, *P*>0.05.

Chronic pressure overload induces a number of structural alterations, not only hypertrophy of cardiomyocytes but also an increase in extracellular matrix (ECM) proteins in the interstitium and perivascular regions of the myocardium [3]. To further determine the effect of Sop on maladaptive cardiac remodeling, PSR staining and quantitative analysis were performed. As expected, hearts subjected to chronic pressure overload developed notably increased fibrosis in the perivascular and interstitial spaces compared with the sham group after 5 weeks of TAC surgery, and the degree of fibrosis was remarkably limited in Sop-treated hearts (Figure 4A,B). Consistently, the expression levels of the markers related to cardiac fibrosis, including Collagen I, Collagen III and connective tissue growth factor (CTGF), decreased significantly in the hearts of Sop-treated mice than those in vehicle group after TAC surgery (Figure 4C–G). Meanwhile, Sop treatment in Sham group had little role in cardiac fibrosis compared with the Sham Saline group (Supplementary Figure S4).

### Sop enhances the decreased autophagy in pathological cardiac hypertrophy

**Figure 5:**
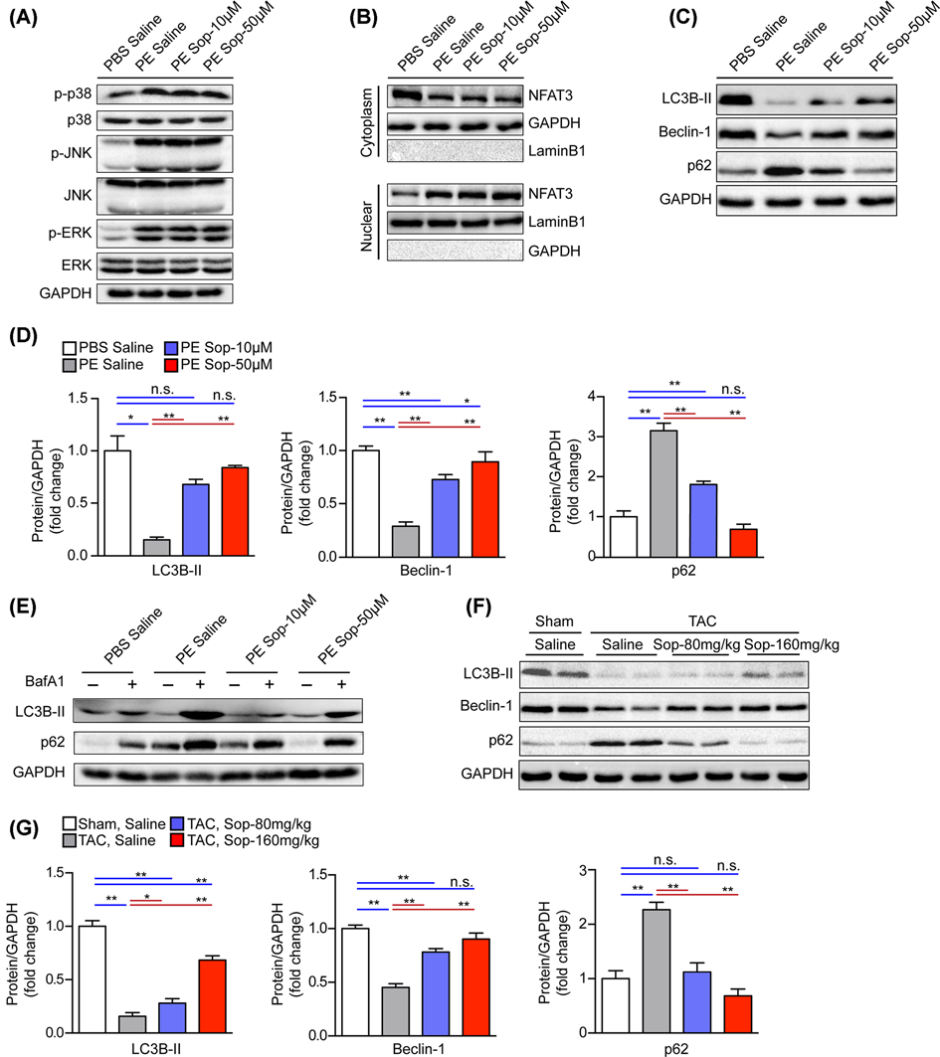
Sop enhances the decreased autophagy in pathological cardiac hypertrophy (**A,B**) Western blot analysis of the phosphorylation levels of (p38, JNK, ERK) and NFAT3 nuclear translocation in indicated group. (**C,D**) Western blot analysis of protein levels and statistical quantification of the autophagy marker proteins (LC3B-II, Beclin-1 and p62) in the NRCMs. Cells treated by PE or PBS in parallel and co-incubated with Sop or Saline treatment for 48 h, *n*=3 independent experiments. (**E**) Protein levels of the autophagy markers proteins (LC3B-II and p62) in the NRCMs. Cells treated by PE or PBS in parallel and co-incubated with Sop or Saline treatment for 48 h in the presence or absence of BafA1. (**F**) Protein levels and statistical quantification of the autophagy marker proteins (LC3B-II, Beclin-1 and p62) from the indicated groups after sham or TAC surgery for 5 weeks, *n*=4 mice per group. GAPDH was used as the internal control in (**A**–**G**). *, *P*<0.05; **, *P*<0.01; n.s., no significance, *P*>0.05. Abbreviation: BafA1, Bafilomycin A1.

PE signaling pathway plays an important role in cardiac hypertrophy, which activates a series of downstream signal events, including activation of NFAT3 nuclear translocation and activation of the three best-characterized mitogen-activated protein kinase (MAPK) subfamilies, namely p38, JNKs and ERKs [22,23]. In order to detect the effect of Sop on MAPK or NFAT3 signal pathway, we treated NRCMs with Sop at different concentrations (10 or 50 μM) for 48 h. Immunoblotting indicated that Sop had no effect on phosphorylation levels of (p38, JNK, ERK) and NFAT3 nuclear translocation (Supplementary Figure S5A,B). Furthmore, Sop treatment under PE stimulation also showed no changes in the phosphorylation levels of (p38, JNK, ERK) and NFAT3 nuclear translocation, compared with corresponding controls, respectively (Figure 5A,B), indicating that Sop did not alleviate cardiac hypertrophy via MAPK or NFAT3 signaling.

Considering the core regulation of autophagy in cardiac hypertrophy, we explored whether Sop rendered cardioprotective function involves autophagy activity and thus measured the protein expression of LC3B-II, p62 and Beclin1. Our results revealed that the protein level of LC3B-II and Beclin1 were largely suppressed in both PE-challenged cardiomyocytes and TAC-insulted mice heart tissues, while p62 was up-regulated in the indicated groups. Notably, Sop treatment significantly increased the expression of LC3B-II and Beclin1, but reduced the accumulation of p62 in NRCMs (Figure 5C,D). In order to detect the effect of Sop on autophagy flux, autophagosome inhibitors Bafilomycin A1 (BafA1) was used in cardiac hypertrophy model in *vitro* [24]. NRCMs were incubated with or without Sop treatment following PE stimulation, BafA1 (100 nM) were added 2 h before harvesting the cells. Immunoblotting showed that Sop promoted the autophagy flux (Figure 5E). The regulation of Sop on autophagy markers (LC3B-II, p62 and Beclin1) was further confirmed in mice after 5 weeks of TAC surgery (Figure 5F,G).

### Sop activates AMPK and inhibits mTORC1 signal pathway

**Figure 6:**
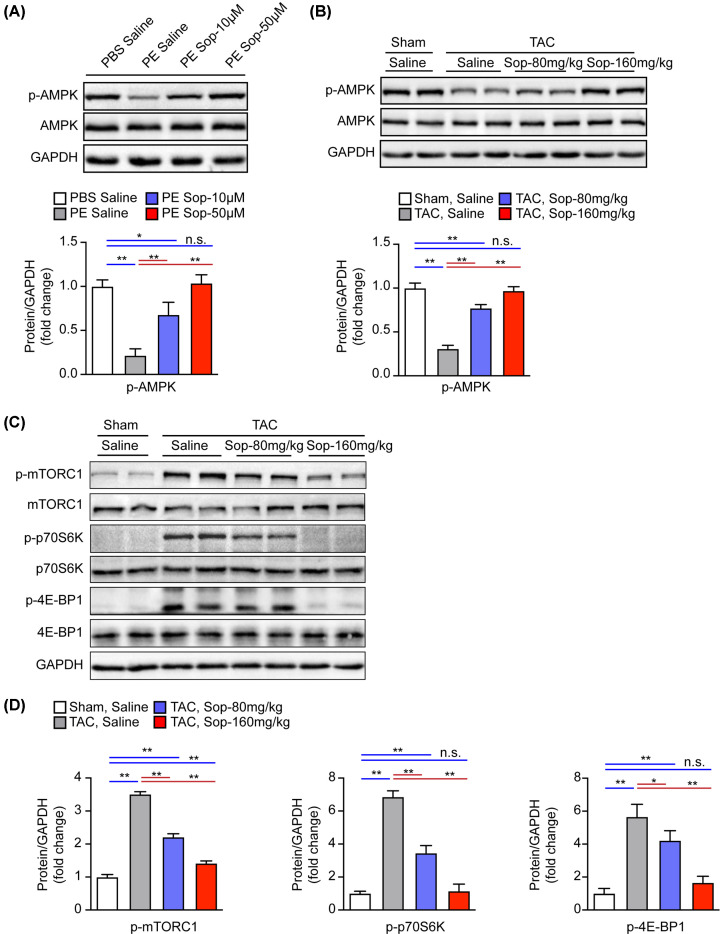
Sop activates AMPK and inhibits mTORC1 signal pathway (**A**) Protein levels and statistical quantification of the phosphorylation of AMPK in the NRCMs. Cells treated by PE or PBS in parallel and co-incubated with Sop or Saline treatment for 48 h, *n*=3 independent experiments. (**B**) Protein levels and statistical quantification of the phosphorylation of AMPK from the indicated groups at 5 weeks after sham or TAC surgery, *n*=4 mice per group. (**C,D**) Protein levels and statistical quantification of the phosphorylation of mTORC1, p70S6K and 4E-BP1 from the indicated groups at 5 weeks after sham or TAC surgery, *n*=4 mice per group. GAPDH was used as the internal control in (A–C). *, *P*<0.05; **, *P*<0.01; n.s., no significance, *P*>0.05.

AMPK-mTORC1 signaling pathway has been considered as an important mechanism in regulating cardiac hypertrophy with the involvement of autophagy [25]. Meanwhile, it is well-known that activation of AMPK significantly decreases the phosphorylation of mTORC1 in heart diseases [26,27]. To further explore the mechanism underlying Sop function on autophagy, we examined the AMPK-mTORC1 signaling pathway by detecting the phosphorylation of AMPK and its downstream targets, including mTORC1, p70S6K and 4E-BP1 in NRCMs and mice heart tissues. Western blots analysis revealed that the compromised AMPK phosphorylation was robustly increased by Sop-treated in both NRCMs and heart tissues (Figure 6A,B). Consistently, the phosphorylation level of mTORC1, p70S6K and 4E-BP1 were remarkably increased after 5 weeks of TAC surgery, which was blunted by Sop treatment (Figure 6C,D). These data suggest that the pro-autophagy effect of Sop may be mediated by the activation of the AMPK/ mTORC1/p70S6K/4E-BP1 axis.

### Sop-attenuated PE-induced NRCMs hypertrophy depends on AMPK activation

**Figure 7:**
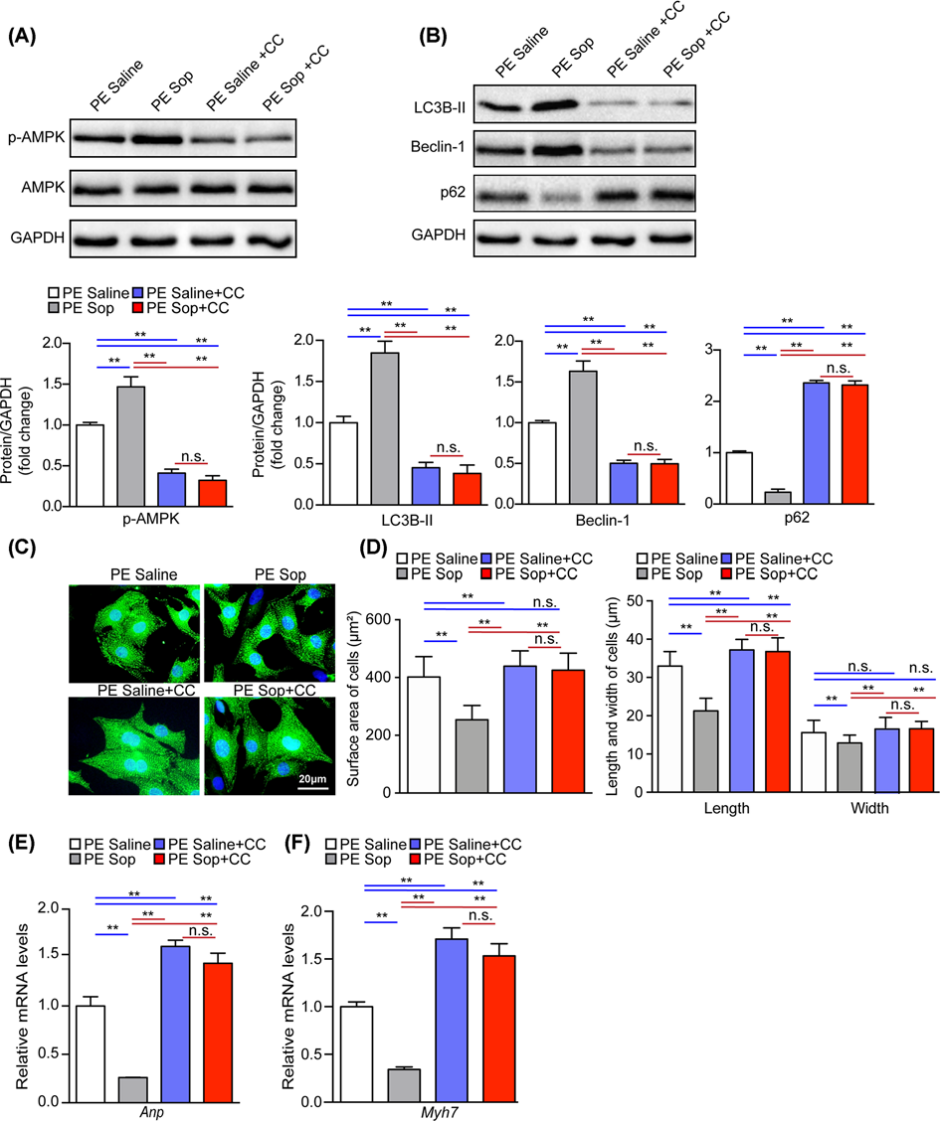
Sop attenuates PE-induced NRCMs hypertrophy depends on AMPK activation (**A**) Protein level and statistical quantification of the phosphorylation of AMPK in the NRCMs. Cells treated by PE and co-incubated with Sop or Saline in the presence or absence of Compound C treatment for 48 h. *n*=3 independent experiments. (**B**) Protein levels and statistical quantification of LC3B-II, Beclin-1, p62 in NRCMs from the indicated groups, *n*=3 independent experiments. (**C**) Representative images of α-actinin (green) and DAPI (blue) stained NRCMs. Cells treated by PE and co-incubated with Sop or Saline in the presence or absence of Compound C treatment for 48 h, (scale bar, 20 µm), *n*=3 independent experiments. (**D**) Statistical quantification of the cell surface area and length and width of NRCMs in the indicated groups, *n*>30 cells per group.*n*=3 independent experiments (**E,F**) mRNA levels of cardiac hypertrophic marker genes (*Anp, Myh7*) in the indicated groups, *n*=3 independent experiments. GAPDH was used as the internal control in (A,B). *, *P*<0.05; **, *P*<0.01; n.s., no significance, *P*>0.05.

To evaluate whether the cardioprotective function of Sop on cardiac hypertrophy is AMPK-dependent, we treated NRCMs with Sop in the presence or absence of AMPK inhibitor compound C. First, we tested the role of compound C in cardiac hypertrophy in *vitro*. NRCMs were treated with compound C at concentrations of (10 and 20 μM) or PBS for 48 h. The results showed that the phosphorylation level of AMPK was decreased significantly by the treatment of compound C in *vitro* (Supplementary Figure S6A). Meanwhile, immunofluorescence staining, cell surface area, cell length and width showed that compound C had no obvious effect on cardiac hypertrophy under base condition (Supplementary Figure S6B,C). However, compound C significantly inhibited AMPK and autophagy activation in both Saline and Sop groups under PE stimulation (Figure 7A,B). Importantly, compound C treatment completely abrogated the effect of Sop on inhibiting cardiomyocyte hypertrophy and the activation of fetal genes (*Anp, Myh7*) (Figure 7C–F). Thus, our findings suggest that Sop negatively modulates pathological cardiac hypertrophy, at least partially, by activating AMPK signaling.

## Discussion

In the present study, we demonstrated that Sop possesses a potent cardioprotective function on pathological cardiac hypertrophy, remodeling and the resultant heart dysfunction. The underlying mechanism of Sop function was largely attributed to the activation of AMPK-mediated autophagy. Considering the wide benefits of Sop on the biological process after AMPK activation and the inhibition of pathological cardiac remodeling, our present data firmly support a clinical translational potential of Sop as an attractive drug for the treatment of pathological cardiac hypertrophy and heart failure.

Traditional Chinese medicines have been widely used in treating cardiovascular diseases [28,29]. Sop is an isoflavone glycoside found in many plant species, which has recently attracted attention because of its anti-inflammation, anti-tumor, anti-obesity, anti-fertility and anti-oxidation activities [30]. Isoflavones are classified as both phytoestrogens and selective estrogen receptor modulators [31]. Additionally, increasing evidences demonstrate that phytoestrogens were considered to protect against several cardiovascular diseases, such as ischemic injury and atherosclerosis [32,33]. Therefore, as an isoflavone, Sop may play a protective role in heart disease.

Our present study yielded several novel findings about the anti-hypertrophic effects of Sop. On one hand, this is the first report that Sop significantly promotes autophagy in cardiac hypertrophy model. Autophagy is associated with a variety of stress reactions in many diseases, including inflammation disease [34], diabetes [35,36] and non-alcoholic fatty liver disease [37,38]. Our study revealed that Sop promoted autophagy in cardiac hypertrophy, which gives us novel insights into the pharmacological potential in previous diseases studies. On the other hand, Sop promoted AMPK activation and negatively regulates mTORC1 signaling pathway. The results of up-regulation of AMPK is consistent with the previous study that Sop increased the phosphorylation of AMPK and promoted lipid metabolism in obesity and type II diabetes [12]. Many isoflavones with phytoestrogenic properties regulate AMPK and play protective roles in cardiac hypertrophy, such as Asiatic acid [39], Geniposide [40] and Indole-3-carbinol [41]. In our study, as an isoflavone with phytoestrogenic properties, Sop does play a protective role in cardiac hypertrophy by promoting the activation of AMPK. Furthermore, the inhibitor of AMPK (compound C) confirmed that Sop promoted autophagy and protected against cardiac hypertrophy, which depended on the activation of AMPK activity.

To date, there are no available pharmacological agents that have been verified to inhibit cardiac hypertrophic development in patients [42,43]. Surgical myectomy and the implantable cardiac defibrillator (ICD) are the only interventions methods in long‐term prognosis [42]. Hence, it remains to find more appropriate drugs that can efficiently and specifically inhibit cardiac hypertrophy. Our study reveals that Sop is a potential cardioprotective compound and robustly attenuates pathological cardiac hypertrophy by activating AMPK-mediated autophagy activation. Therefore, our study expands the function and possible clinical application of Sop for cardiac hypertrophy and heart failure, which also represents an advanced utilization of a natural product for more severe heart diseases.

Inevitably, the limitations of our study should be noted. First, we need to add more models of cardiac hypertrophy to verify the protective effect of Sop. Second, the specific mechanism by which Sop activates AMPK remains to be further explored; and finally, the molecular target by which Sop plays a protective role in cardiac hypertrophy remains to be further confirmed.

## Perspectives

- The present study is the first time to demonstrate that Sop protects against pathological cardiac hypertrophy.
- Sop treatment induced a remarkable activation of AMPK/mTORC1-autophagy cascade following sustained hypertrophic stimuli.
- These findings broaden our understanding of the clinical translational potential of Sop and firmly supported Sop as an attractive drug for the treatment of pathological cardiac hypertrophy and heart failure.

## Supplementary Material

Supplementary Figures S1-S7 and Tables S1-S2

## Abbreviations

ANP: A-type natriuretic peptide
AMPK: AMP-activated kinase
BafA1: Bafilomycin A1
BNP: B-type natriuretic peptide
CSA: cross-sectional area
DAPI: 4′,6-diamidino-2-phenylindole
FS: fractional shortening
GAPDH: glyceraldehyde-3-phosphate dehydrogenase
H&E: Hematoxylin and Eosin
HW: heart weight
HW/BW: heart weight/body weight
HW/TL: heart weight/tibia length
LVEDd: left ventricular end-diastolic diameter
LVESd: left ventricular end-systolic diameter
LW/BW: lung weight/body weight
MYH7: myosin heavy chain 7
NFAT3: nuclear factor of activated T-cells 3
NRCM: neonatal rat cardiomyocyte
p70S6K: Ribosomal Protein S6 Kinase B1
PE: phenylephrine
PSR: Picrosirius Red
Sop: sophoricoside
SPF: Specified Pathogen Free
TAC: transverse aortic constriction
4E-BP1: Eukaryotic Translation Initiation Factor 4E Binding Protein 1

## References

[1] Tanai E. and Frantz S. (2015) Pathophysiology of heart failure. Compr. Physiol. 6, 187–214 10.1002/cphy.c140055. 26756631

[2] Mosterd A. and Hoes A.W. (2007) Clinical epidemiology of heart failure. Heart 93, 1137–1146 17699180 10.1136/hrt.2003.025270 PMC1955040

[3] Creemers E.E. and Pinto Y.M. (2011) Molecular mechanisms that control interstitial fibrosis in the pressure-overloaded heart. Cardiovasc. Res. 89, 265–272 20880837 10.1093/cvr/cvq308

[4] Shimizu I. and Minamino T. (2016) Physiological and pathological cardiac hypertrophy. J. Mol. Cell Cardiol. 97, 245–262 27262674 10.1016/j.yjmcc.2016.06.001

[5] Doroudgar S. and Glembotski C.C. (2011) The cardiokine story unfolds: ischemic stress-induced protein secretion in the heart. Trends Mol. Med. 17, 207–214 10.1016/j.molmed.2010.12.003 21277256 PMC3078974

[6] Li L. et al. (2016) The role of autophagy in cardiac hypertrophy. Acta Biochim. Biophys. Sin. (Shanghai) 48, 491–500 10.1093/abbs/gmw025 27084518 PMC4913516

[7] Maillet M., van Berlo J.H. and Molkentin J.D. (2013) Molecular basis of physiological heart growth: fundamental concepts and new players. Nat. Rev. Mol. Cell Biol. 14, 38–48 10.1038/nrm3495 23258295 PMC4416212

[8] De Meyer G.R. and De Keulenaer G.W. Martinet W. (2010) Role of autophagy in heart failure associated with aging. Heart Fail. Rev. 15, 423–430 10.1007/s10741-010-9166-6 20383579

[9] Schiattarella G.G. and Hill J.A. (2016) Therapeutic targeting of autophagy in cardiovascular disease. J. Mol. Cell Cardiol. 95, 86–93 10.1016/j.yjmcc.2015.11.019 26602750 PMC4871782

[10] Wang X. and Cui T. (2017) Autophagy modulation: a potential therapeutic approach in cardiac hypertrophy. Am. J. Physiol. Heart Circ. Physiol. 313, H304–H319 10.1152/ajpheart.00145.2017 28576834 PMC5582915

[11] Li W. and Lu Y. (2018) Hepatoprotective effects of Sophoricoside against fructose-induced liver injury via regulating lipid metabolism, oxidation, and inflammation in mice. J. Food Sci. 83, 552–558 10.1111/1750-3841.14047 29350757

[12] Wu C. et al. (2013) Modulation of lipogenesis and glucose consumption in HepG2 cells and C2C12 myotubes by sophoricoside. Molecules 18, 15624–15635 10.3390/molecules181215624 24352018 PMC6270613

[13] Xing C. et al. (2019) New crystal forms and amorphous phase of sophoricoside: X-ray structures and characterization. R. Soc. Open Sci. 6, 181905 10.1098/rsos.18190530800407 30800407 PMC6366211

[14] Lee H.K. et al. (2013) Sophoricoside isolated from Sophora japonica ameliorates contact dermatitis by inhibiting NF-kappaB signaling in B cells. Int. Immunopharmacol. 15, 467–473 10.1016/j.intimp.2013.01.025 23415872

[15] Kim S.J. et al. (2013) The ameliorative effect of sophoricoside on mast cell-mediated allergic inflammation in vivo and in vitro. Molecules 18, 6113–6127 10.3390/molecules18056113 23698058 PMC6270464

[16] Lou J. et al. (2016) Type III transforming growth factor-β receptor drives cardiac hypertrophy through β-arrestin2–dependent activation of calmodulin-dependent protein kinase II. Hypertension 68, 654–666 10.1161/HYPERTENSIONAHA.116.07420 27432858

[17] Jiang D.S. et al. (2014) IRF8 suppresses pathological cardiac remodelling by inhibiting calcineurin signalling. Nat. Commun. 5, 3303 10.1038/ncomms4303 24526256 PMC3929801

[18] Jiang D.S. et al. (2014) Interferon regulatory factor 7 functions as a novel negative regulator of pathological cardiac hypertrophy. Hypertension 63, 713–722 10.1161/HYPERTENSIONAHA.113.02653 24396025 PMC5349187

[19] Ji Y.X. et al. (2016) The ubiquitin E3 ligase TRAF6 exacerbates pathological cardiac hypertrophy via TAK1-dependent signalling. Nat. Commun. 7, 11267 10.1038/ncomms11267 27249171 PMC4895385

[20] Li H. et al. (2010) Regulator of G protein signaling 5 protects against cardiac hypertrophy and fibrosis during biomechanical stress of pressure overload. Proc. Natl. Acad. Sci. U.S.A. 107, 13818–13823 10.1073/pnas.1008397107 20643937 PMC2922261

[21] Jiang D.S. et al. (2013) Role of interferon regulatory factor 4 in the regulation of pathological cardiac hypertrophy. Hypertension 61, 1193–1202 10.1161/HYPERTENSIONAHA.111.00614 23589561 PMC3734933

[22] Zong J. et al. (2018) Nuclear localization leucine-rich-repeat protein 1 deficiency protects against cardiac hypertrophy by pressure overload. Cell. Physiol. Biochem. 48, 75–86 10.1159/000491664 30001530

[23] van Berlo J.H., Maillet M. and Molkentin J.D. (2013) Signaling effectors underlying pathologic growth and remodeling of the heart. J. Clin. Invest. 123, 37–45 10.1172/JCI62839 23281408 PMC3533272

[24] Mizushima N., Yoshimori T. and Levine B. (2010) Methods in mammalian autophagy research. Cell 140, 313–326 10.1016/j.cell.2010.01.028 20144757 PMC2852113

[25] Feng Y., Zhang Y. and Xiao H. (2018) AMPK and cardiac remodelling. Sci. China Life Sci. 61, 14–23 10.1007/s11427-017-9197-5 29170891

[26] Chen W. et al. (2019) Correction to: Cryptotanshinone activates AMPK-TSC2 axis leading to inhibition of mTORC1 signaling in cancer cells. BMC Cancer 19, 257 10.1186/s12885-019-5458-y 30902078 PMC6429697

[27] Holczer M. et al. (2019) A double negative feedback loop between mTORC1 and AMPK kinases guarantees precise autophagy induction upon cellular stress. Int. J. Mol. Sci. 20, 5543 10.3390/ijms20225543 31703252 PMC6888297

[28] Zhang C. et al. (2011) Mitochondrial dysfunction induced by excessive ROS/RNS-metabolic cardiovascular disease and traditional Chinese medicines intervention. Zhongguo Zhong Yao Za Zhi 36, 2423–2428 22121816

[29] Zhang Q.F. et al. (2001) Study of cardiovascular and cerebrovascular disease in Chinese traditional medicines on Pb, Cr, Cd by AAS. Guang Pu Xue Yu Guang Pu Fen Xi 21, 865–867 12958918

[30] Gabor M. (1961) The hormonal effect of an isoflavone derivative (sophoricoside). Kiserl Orvostud. 13, 133–134 13702797

[31] Messina M. (2016) Soy and health update: evaluation of the clinical and epidemiologic literature. Nutrients 8, 754 10.3390/nu8120754 27886135 PMC5188409

[32] Huang Z. and Liu Y. Huang X. (2018) Formononetin may protect aged hearts from ischemia/reperfusion damage by enhancing autophagic degradation. Mol. Med. Rep. 6, 4821–4830 10.3892/mmr.2018.9544 PMC6236296 30320398

[33] Myasoedova V.A. et al. (2016) Anti-atherosclerotic effects of a phytoestrogen-rich herbal preparation in postmenopausal women. Int. J. Mol. Sci. 17, 1318 10.3390/ijms17081318 27529226 PMC5000715

[34] Wang X. et al. (2016) Roseotoxin B improves allergic contact dermatitis through a unique anti-inflammatory mechanism involving excessive activation of autophagy in activated T lymphocytes. J. Invest. Dermatol. 136, 1636–1646 10.1016/j.jid.2016.04.017 27155460

[35] Demirtas L. et al. (2016) Apoptosis, autophagy & endoplasmic reticulum stress in diabetes mellitus. Indian J. Med. Res. 144, 515–524 28256459 10.4103/0971-5916.200887 PMC5345297

[36] Ji J. et al. (2019) Type 2 diabetes is associated with suppression of autophagy and lipid accumulation in beta-cells. J. Cell. Mol. Med. 23, 2890–2900 10.1111/jcmm.14172 30710421 PMC6433726

[37] Mao Y. et al. (2016) Autophagy: a new target for nonalcoholic fatty liver disease therapy. Hepat. Med. 8, 27–37 10.2147/HMER.S98120 27099536 PMC4822806

[38] Li X.Q. et al. (2016) Autophagy and nonalcoholic fatty liver disease. Zhonghua Gan Zang Bing Za Zhi 24, 632–635 27788715 10.3760/cma.j.issn.1007-3418.2016.08.016 PMC12769676

[39] Ma Z.G. et al. (2016) Asiatic acid protects against cardiac hypertrophy through activating AMPKalpha signalling pathway. Int. J. Biol. Sci. 12, 861–871 10.7150/ijbs.14213 27313499 PMC4910604

[40] Ma Z.G. et al. (2016) Protection against cardiac hypertrophy by geniposide involves the GLP-1 receptor/AMPKalpha signalling pathway. Br. J. Pharmacol. 173, 1502–1516 10.1111/bph.13449 26845648 PMC4831312

[41] Deng W. et al. (2013) Indole-3-carbinol protects against pressure overload induced cardiac remodeling via activating AMPK-alpha. Mol. Nutr. Food Res. 57, 1680–1687 10.1002/mnfr.201300012 23625645

[42] Spoladore R. et al. (2012) Pharmacological treatment options for hypertrophic cardiomyopathy: high time for evidence. Eur. Heart J. 33, 1724–1733 10.1093/eurheartj/ehs150 22719025

[43] Axelsson A. et al. (2015) Efficacy and safety of the angiotensin II receptor blocker losartan for hypertrophic cardiomyopathy: the INHERIT randomised, double-blind, placebo-controlled trial. Lancet Diabetes Endocrinol. 3, 123–131 10.1016/S2213-8587(14)70241-4 25533774

