# Supplementary material for: Sophoricoside ameliorates cardiac hypertrophy by activating AMPK/mTORC1-mediated autophagy
Source: Biosci Rep. 2020 Nov 19;40(11):BSR20200661. doi: 10.1042/BSR20200661 (PMC7677750; doi:10.1042/BSR20200661)
Supplement: Supplementary Figures S1-S7 and Tables S1-S2 [file BSR-2020-0661_supp.pdf]

FigureS1

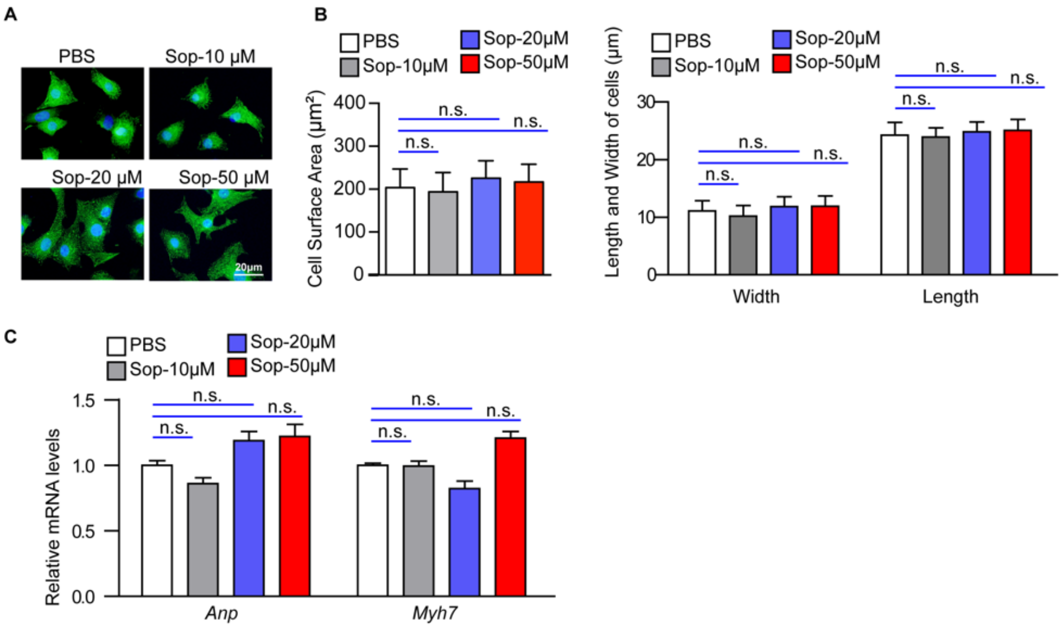

Supplementary Fig S1. The effect of Sop in different concentrations on cardiac hypertrophy in NRCMs *in vitro*. (A) Representative images of α-actinin (green) and DAPI (blue) stained NRCMs that were treated by Sop or PBS for 48 h. Scale bar, 20 μm. (B) Statistical quantification of surface area as well as the length and width of NRCMs in the indicated groups. $n > 50$ cells per group. (C) mRNA levels of hypertrophic marker genes (*Anp*, *Myh7*) in NRCMs in the indicated groups. n.s., no significance, $P > 0.05$.

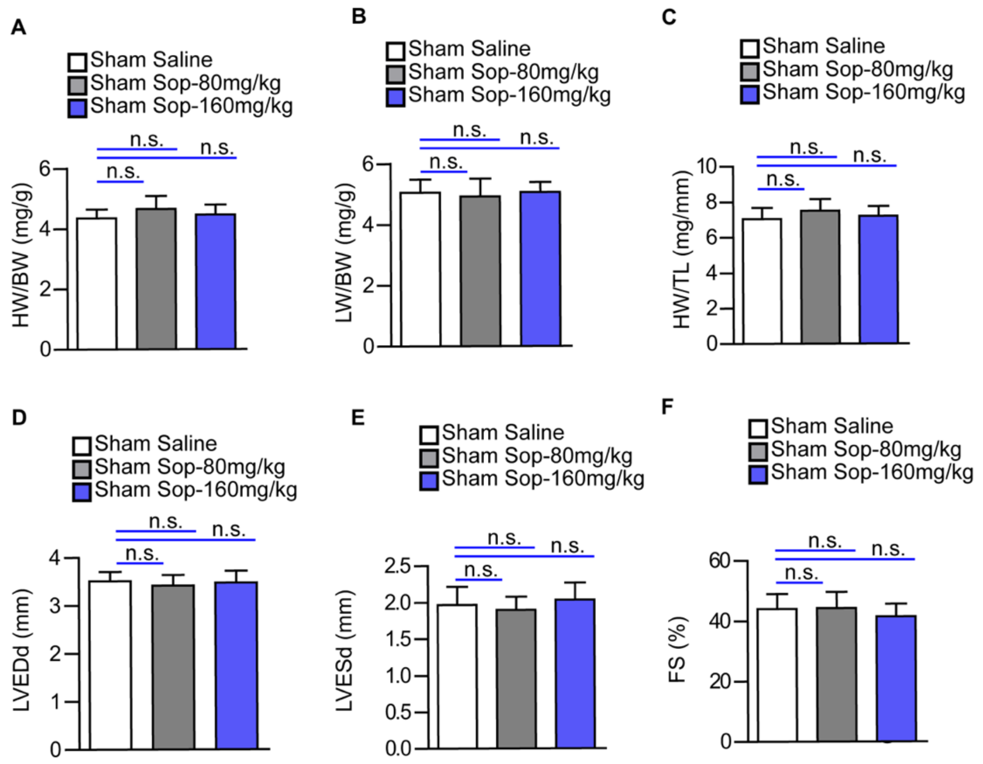

Supplementary Fig S2. The effect of Sop in different concentrations and cardiac function in mice *in vivo*. (A-C) Comparison of the (A) heart weight to body weight (HW/BW), (B) Lung weight to body weight (LW/BW) and (C) Heart weight to tibia length (HW/TL) ratios of mice in different groups after sham surgery. *n*=10 mice for per group. (D-F) Echocardiography results for the measurement of myocardial function (LVEDd, LVESd and FS) in the indicated groups. *n*=10 mice for per group. n.s., no significance, $P > 0.05$.

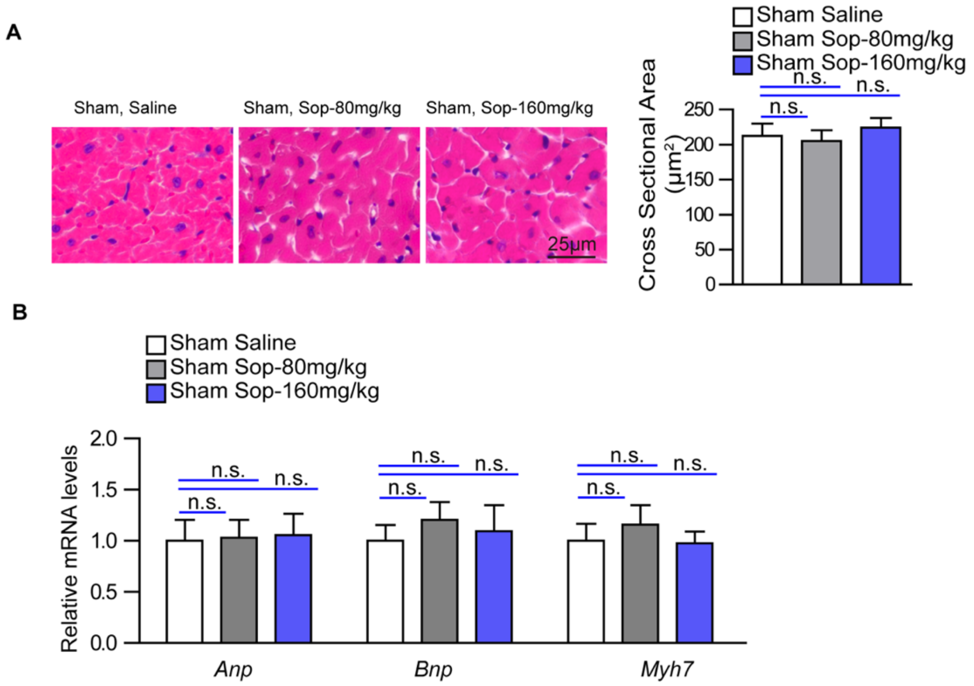

Supplementary Fig S3. The effect of Sop in different concentrations on cardiac hypertrophy in mice *in vivo*. (A) Representative heart sections with H&E staining (scale bar, 25 μm) and quantification of the CSA of cardiomyocytes from the indicated groups. *n*=6 mice for per group. (B) mRNA levels of hypertrophic marker genes (*Anp, Bnp, Myh7*). *n*=4 mice for per group. n.s., no significance, $P > 0.05$.

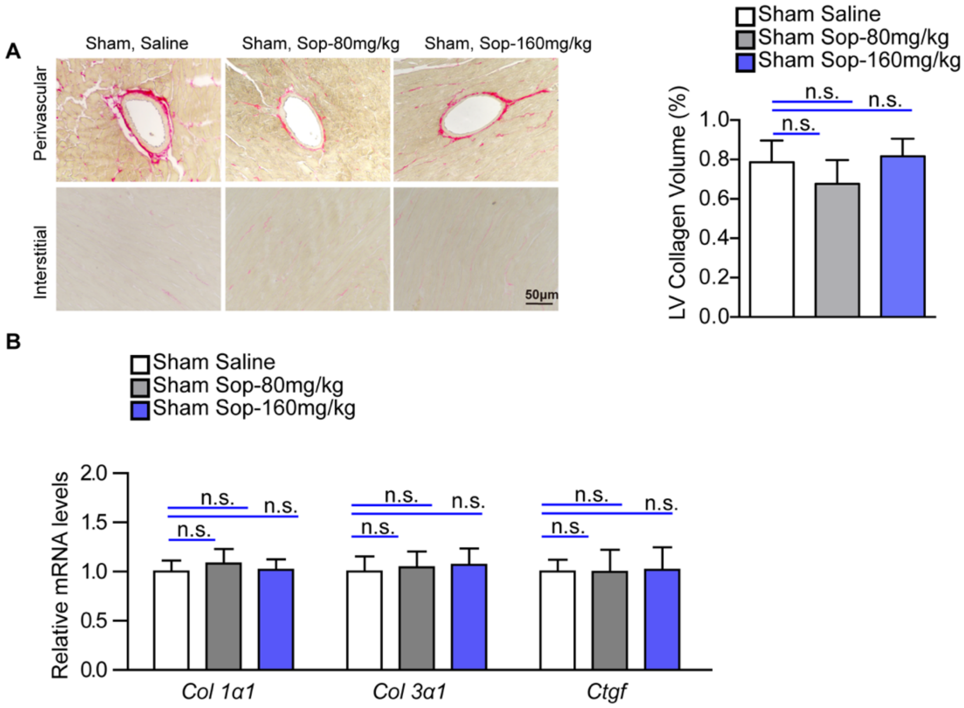

Supplementary Fig S4. The effect of Sop in different concentrations on cardiac fibrosis in mice *in vivo*. (A) Histological analysis of hearts sections with PSR staining (scale bar, 50 μm) and the quantification of the LV-collagen volume in the indicated groups. *n*=6 mice for per group. (B) mRNA levels of fibrotic marker genes (*col1α1*, *col3α1*, ctgf) in the indicated groups. *n*=4 mice for per group. n.s., no significance, $P > 0.05$.

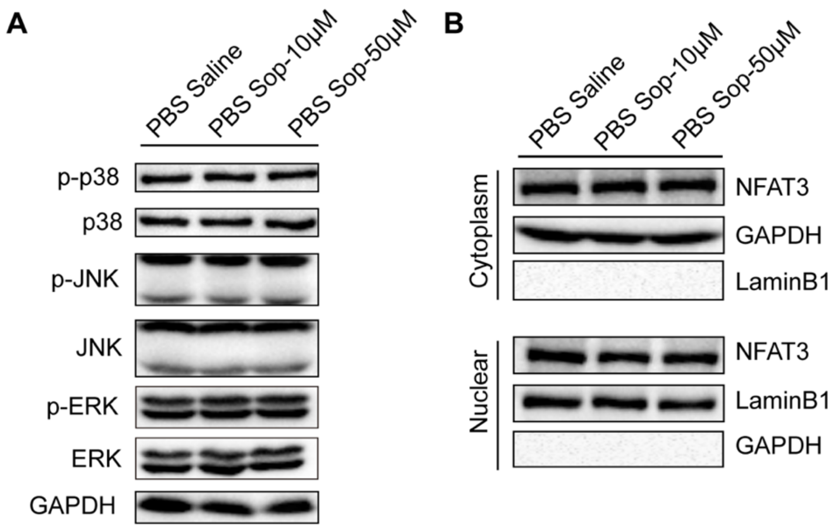

Supplementary Fig S5. The effect of Sop in different concentrations on PE signal pathway in NRCMs *in vitro*. (A) Protein levels of the phosphorylation levels of (p38, JNK, ERK) and NFAT3 in NRCMs that were treated by Sop or Saline treatment for 48 h at baseline. GAPDH was used as the loading control. (B) The protein level of NFAT3 in cytoplasm and nuclear of NRCMs treated with saline or Sop for 48 h at baseline.

Figure S6

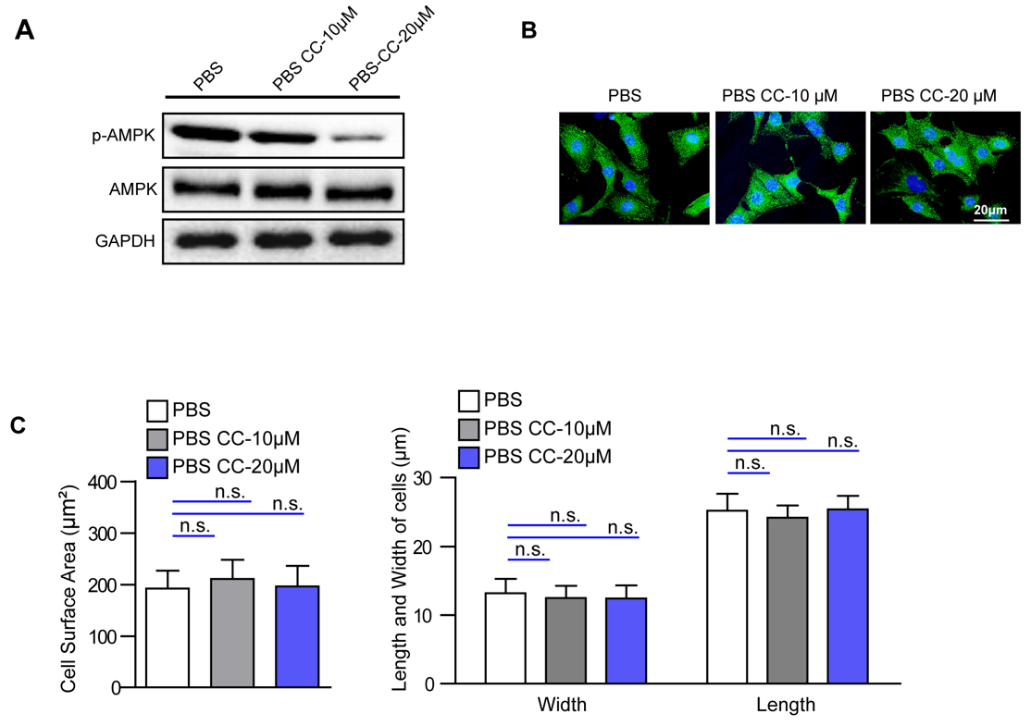

Supplementary Fig S6. The effect of compound C in different concentrations on cardiac hypertrophy in NRCMs *in vitro*. (A) Protein levels and statistical quantification of the phosphorylation of AMPK in the NRCMs treated with compound C or saline treatment for 48 h. (B) Representative images of α-actinin (green) and DAPI (blue) stained NRCMs with PBS treatment in the presence or absence of compound C for 48 h. scale bar, 20 μm. (C) Statistical quantification of the cell surface area and length and width of NRCMs in the indicated groups. $n > 50$ cells per group. n.s., no significance, $P > 0.05$.

Western blots for Fig.1E

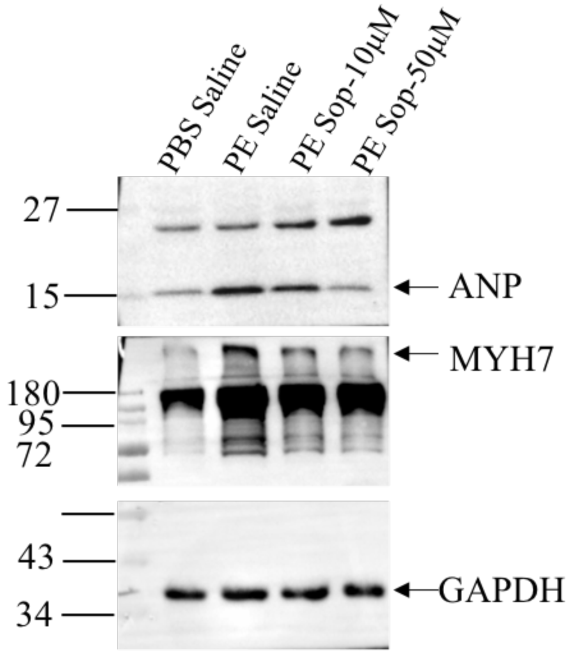

Supplementary Fig S7. Original uncropped gels of representative Western blot images relating to indicated Figures

Western blots for Fig.3G

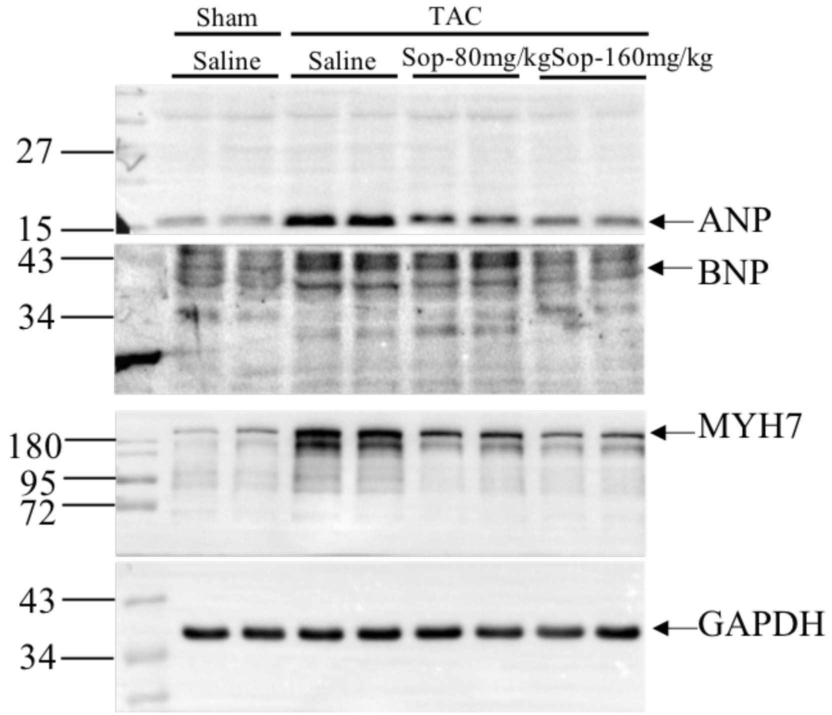

Supplementary Fig S7. Original uncropped gels of representative Western blot images relating to indicated Figures

Western blots for Fig.4F

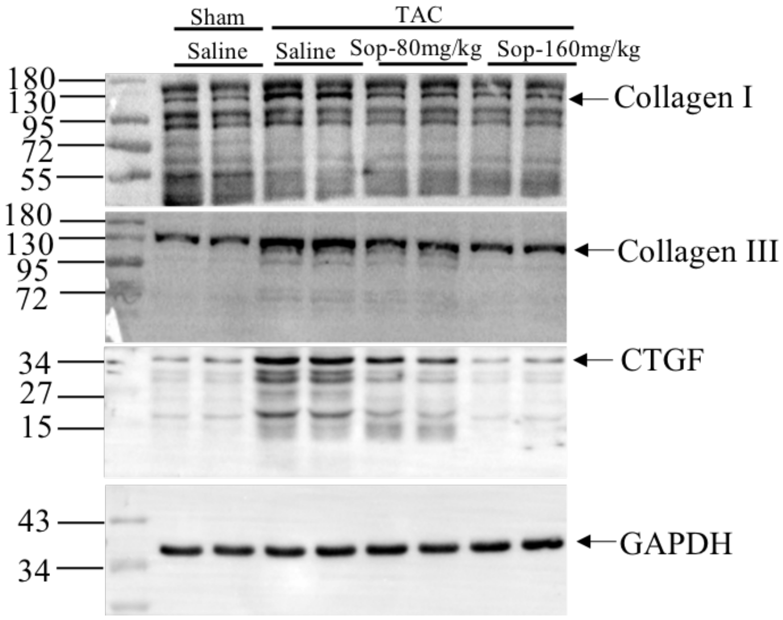

Supplementary Fig S7. Original uncropped gels of representative Western blot images relating to indicated Figures

Western blots for Fig.5A

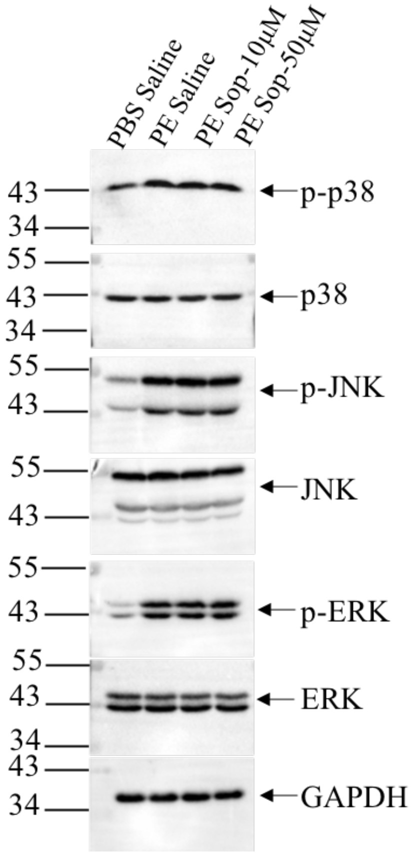

Supplementary Fig S7. Original uncropped gels of representative Western blot images relating to indicated Figures

Western blots for Fig.5B

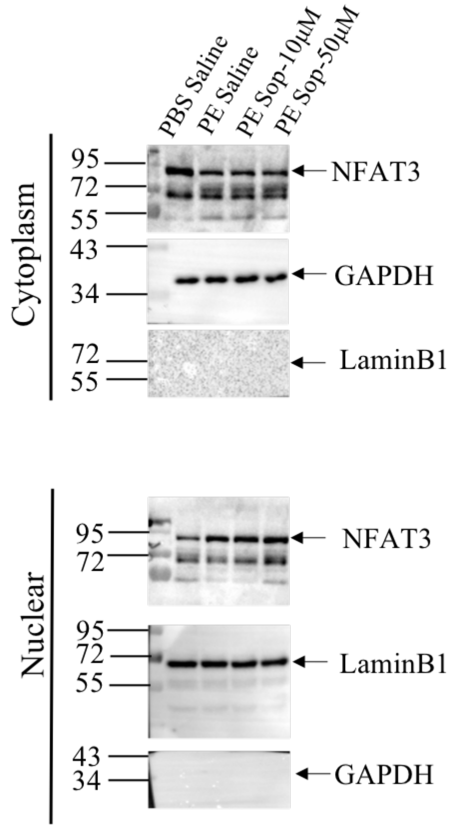

Supplementary Fig S7. Original uncropped gels of representative Western blot images relating to indicated Figures

Western blots for Fig.5C

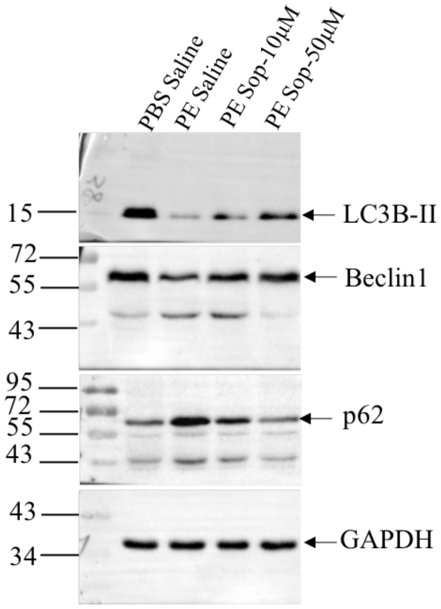

Supplementary Fig S7. Original uncropped gels of representative Western blot images relating to indicated Figures

Western blots for Fig.5E

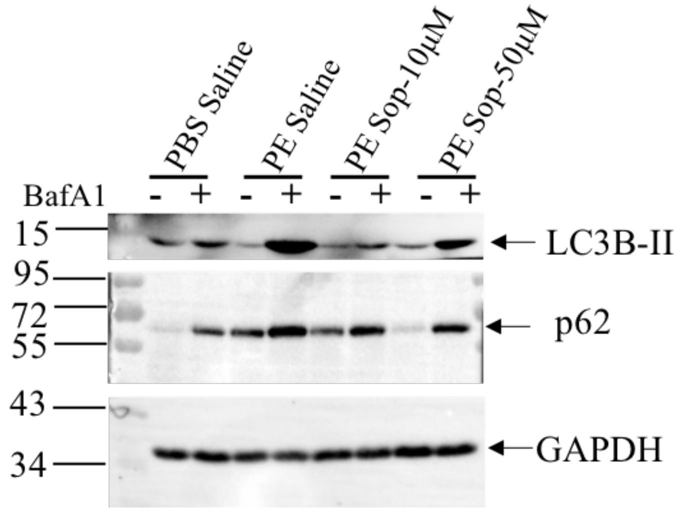

Supplementary Fig S7. Original uncropped gels of representative Western blot images relating to indicated Figure

Western blots for Fig.5F

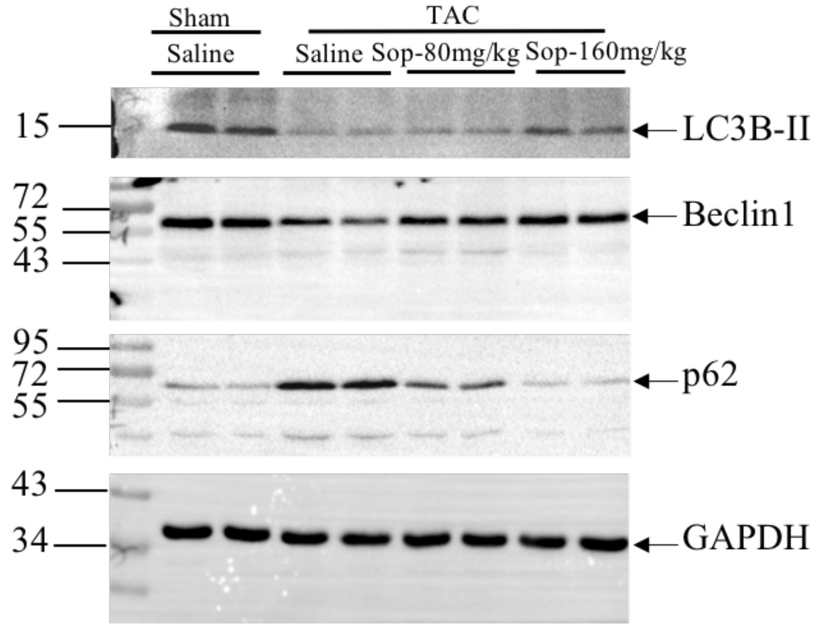

Supplementary Fig S7. Original uncropped gels of representative Western blot images relating to indicated Figure

Western blots for Fig.6A

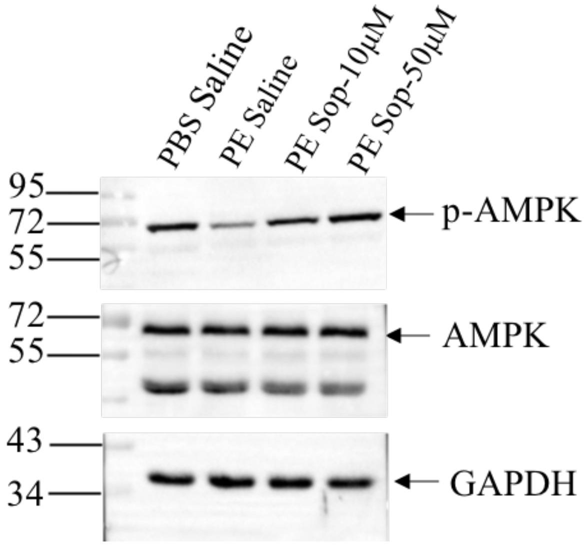

Supplementary Fig S7. Original uncropped gels of representative Western blot images relating to indicated Figure

Western blots for Fig.6B

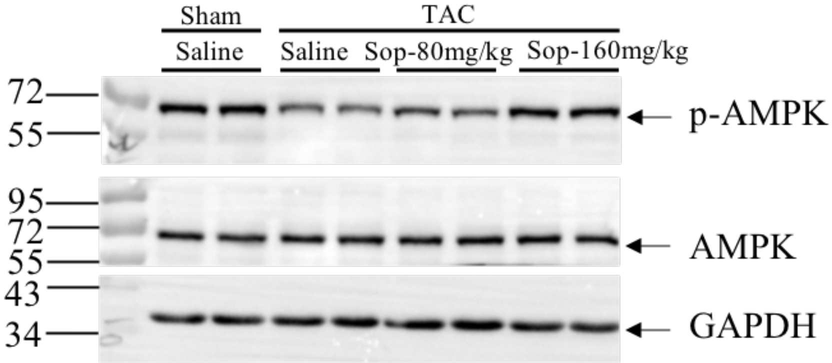

Supplementary Fig S7. Original uncropped gels of representative Western blot images relating to indicated Figure

Western blots for Fig.6C

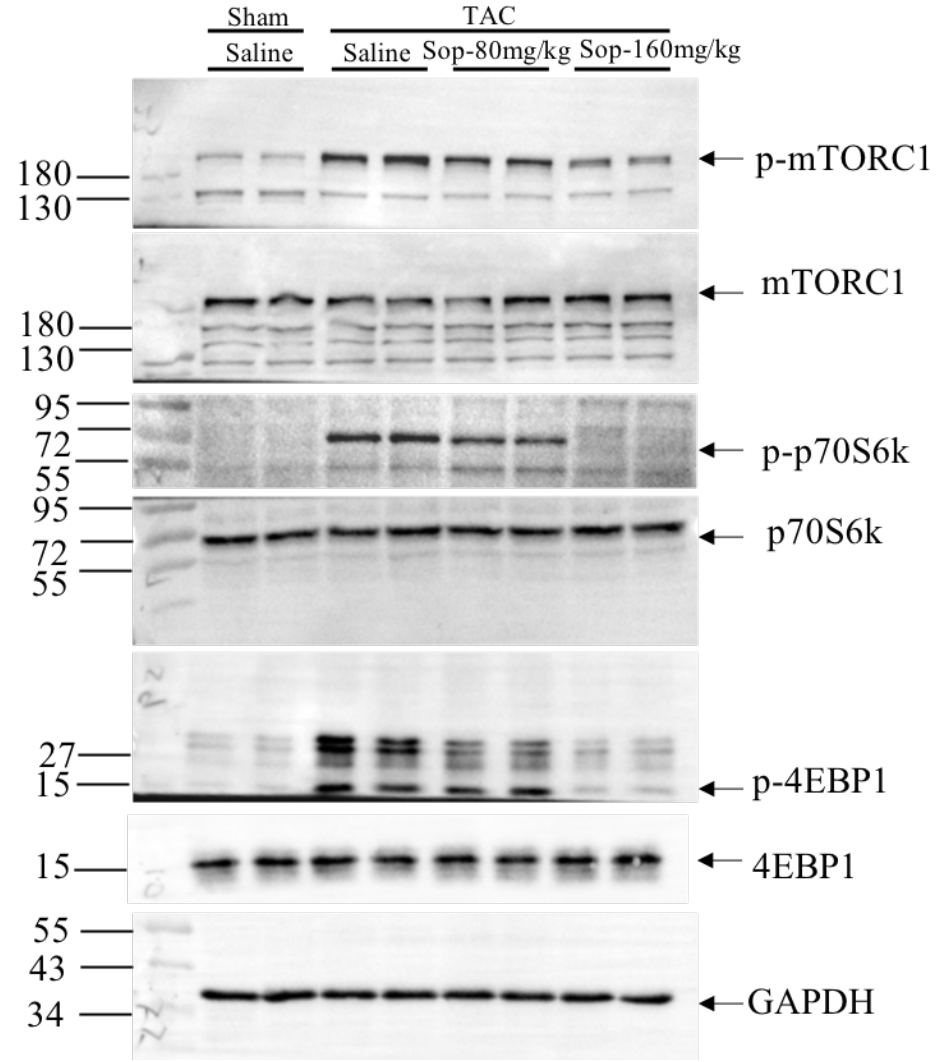

Supplementary Fig S7. Original uncropped gels of representative Western blot images relating to indicated Figure

Western blots for Fig.7A

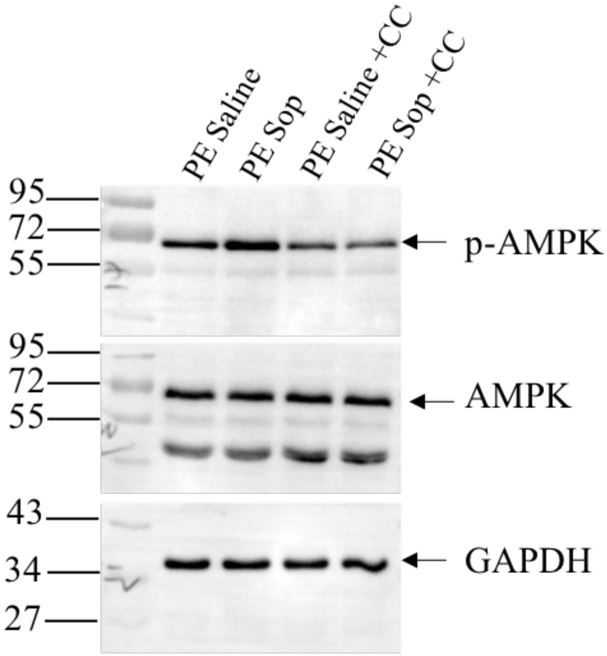

Supplementary Fig S7. Original uncropped gels of representative Western blot images relating to indicated Figures

Western blots for Fig.7B

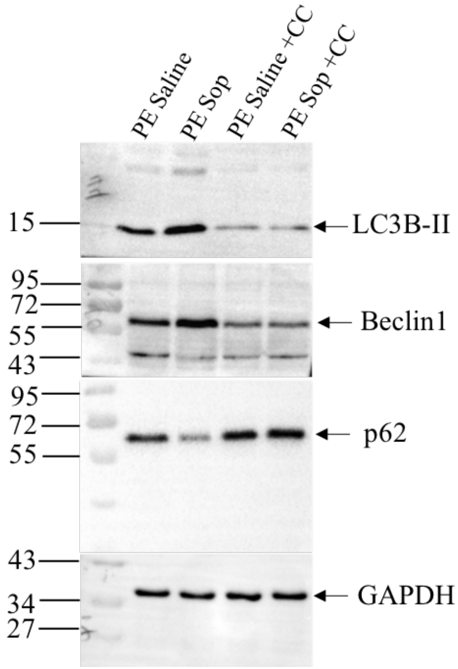

Supplementary Fig S7. Original uncropped gels of representative Western blot images relating to indicated Figure

Western blots for Fig.S5A

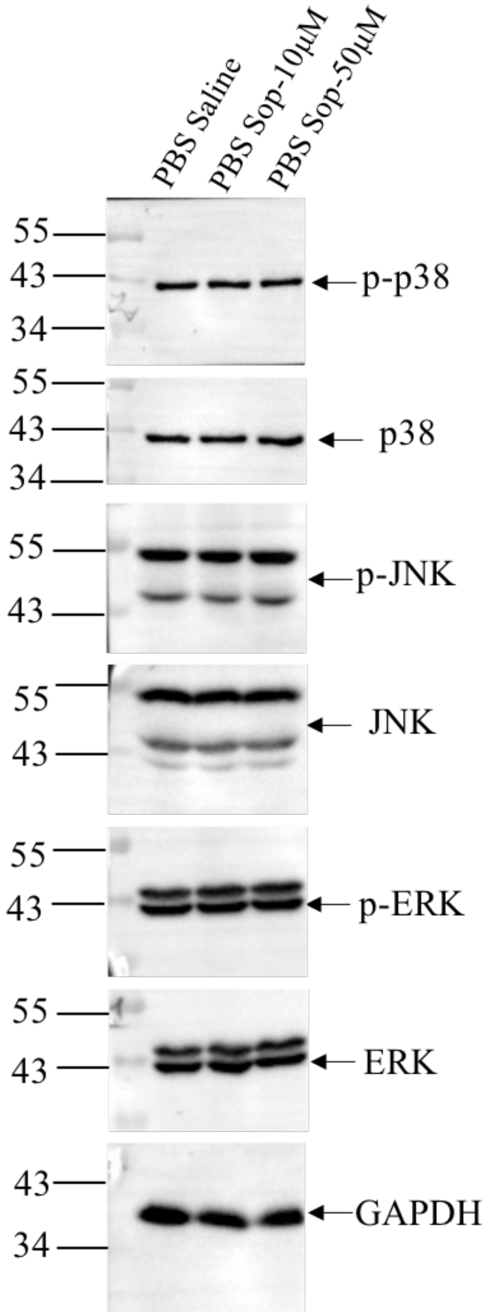

Supplementary Fig S7. Original uncropped gels of representative Western blot images relating to indicated Figures

Western blots for Fig.S5B

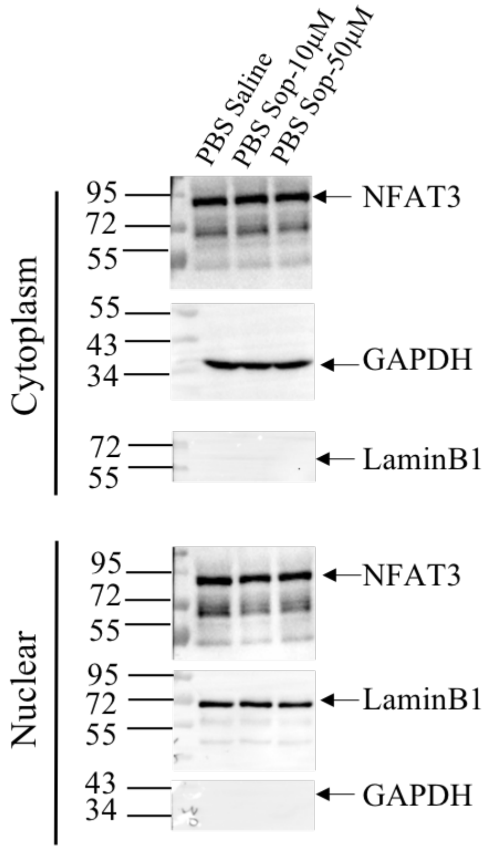

Supplementary Fig S7. Original uncropped gels of representative Western blot images relating to indicated Figures

Western blots for Fig.S6A

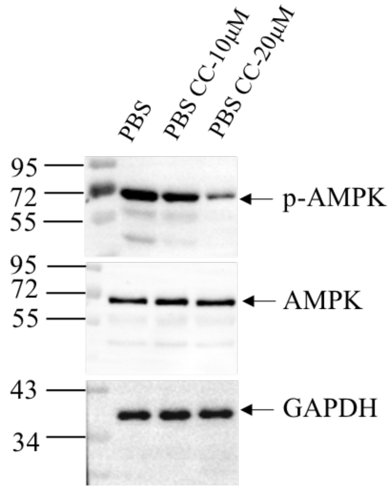

Supplementary Fig S7. Original uncropped gels of representative Western blot images relating to indicated Figures

Supplementary Table 1 The information of antibodies used in Western blotting

Antibody	Manufacturer	Catalogue number	Dilution ratio
ANP	ABclonal	A1609	1:1000
BNP	Santa Cruz Biotechnology	sc-271185	1:200
MYH7	Santa Cruz Biotechnology	sc-53090	1:200
Collagen I	Santa Cruz Biotechnology	sc-293182	1:1000
Collagen III	Santa Cruz Biotechnology	sc-271249	1:1000
CTGF	Santa Cruz Biotechnology	sc-14939	1:200
LC3B-II	Cell signaling Technology	3868	1:1000
p62	Cell signaling Technology	5114	1:1000
Beclin1	Cell signaling Technology	3495	1:1000
p-AMPK	Cell signaling Technology	2535	1:1000
AMPK	Cell signaling Technology	2603	1:1000
p-4EBP1	Cell signaling Technology	2855	1:1000
4EBP1	Cell signaling Technology	9644	1:1000
p-p70S6k	Cell signaling Technology	9208	1:1000
p70S6k	Cell signaling Technology	2708	1:1000
p-ERK	Cell signaling Technology	4370	1:1000
ERK	Cell signaling Technology	4695	1:1000
p-JNK	Cell signaling Technology	4668	1:1000
JNK	Cell signaling Technology	9252	1:1000
p-p38	Cell signaling Technology	4511	1:1000
p38	Cell signaling Technology	9212	1:1000
p-mTORC	Cell signaling Technology	2971	1:1000
mTORC	Cell signaling Technology	2983	1:1000
NFAT3	Sigma	SAB4501982	1:1000
LaminB1	ABCAM	ab16048	1:1000
GAPDH	Cell signaling Technology	5174	1:1000

Supplementary Table 2 The information of Primers used in Quantitative real-time PCR (qRT-PCR)

Gene name	Forward primer (mouse)	Reverse primer (mouse)
Anp	TCGGAGCCTACGAAGATCCA	TTCGGTACCGGAAGCTGTTG
Bnp	GAAGGACCAAGGCCTCACAA	TTCAGTGCGTTACAGCCCAA
Myh7	CAACCTGTCCAAGTTCCGCA	TACTCCTCATTCAGGCCCTTG
Collagen Iα	TGCTAACGTGGTTCGTGACCGT	ACATCTTGAGGTCGCGGCATGT
Collagen IIIα	ACGTAAGCACTGGTGGACAG	CCGGCTGGAAAGAAGTCTGA
Ctgf	TGACCCCTGCGACCCACA	TACACCGACCCACCGAAGACACAG
Gapdh	ACTCCACTCACGGCAAATTC	TCTCCATGGTGGTGAAGACA

Gene name	Forward primer (rat)	Reverse primer (rat)
Anp	AAAGCAAACTGAGGGCTCTGCT	TTCGGTACCGGAAGCTGTTGCA
Myh7	AGTGAAGAGCCTCCAGAGTTTG	GTTGATGAGGCTGGTGTTCTGG
Gapdh	TGTGAACGGATTTGGCCCTA	GATGGTGATGGGTTTCCCGT
